# A Novel H_2_O_2_ Generator for Tumor Chemotherapy-Enhanced CO Gas Therapy

**DOI:** 10.3389/fonc.2021.738567

**Published:** 2021-09-21

**Authors:** Yang Li, Zeming Liu, Weng Zeng, Ziqi Wang, Chunping Liu, Ning Zeng, Keli Zhong, Dazhen Jiang, Yiping Wu

**Affiliations:** ^1^ Department of Plastic Surgery, Tongji Hospital, Tongji Medical College, Huazhong University of Science and Technology, Wuhan, China; ^2^ Department of Gastrointestinal Surgery, Shenzhen People’s Hospital (The Second Clinical Medical College, Jinan University, The First Affiliated Hospital, Southern University of Science and Technology), Shenzhen, China; ^3^ Department of Ophthalmology, Zhongnan Hospital of Wuhan University, Wuhan, China; ^4^ Key Laboratory of Artificial Micro- and Nano-Structures of Ministry of Education, School of Physics and Technology, Wuhan University, Wuhan, China; ^5^ Department of Radiation and Medical Oncology, Hubei Key Laboratory of Tumor Biological Behaviors, Hubei Cancer Clinical Study Center, Zhongnan Hospital of Wuhan University, Wuhan, China

**Keywords:** H_2_O_2_ generator, CO gas therapy, camptothecin, ZIF-8 nanoparticles, TME (tumor microenvironment)

## Abstract

Carbon monoxide (CO) gas therapy is a promising cancer treatment. However, gas delivery to the tumor site remains problematic. Proper tunable control of CO release in tumors is crucial to increasing the efficiency of CO treatment and reducing the risk of CO poisoning. To overcome such challenges, we designed ZCM, a novel stable nanotechnology delivery system comprising manganese carbonyl (MnCO) combined with anticancer drug camptothecin (CPT) loaded onto a zeolitic imidazole framework-8 (ZIF-8). After intravenous injection, ZCM gradually accumulates in cancerous tissues, decomposing in the acidic tumor microenvironment, releasing CPT and MnCO. CPT acts as a chemotherapy agent destroying tumors and producing copious H_2_O_2_. MnCO can react with the H_2_O_2_ to generate CO, powerfully damaging the tumor. Both *in vitro* and *in vivo* experiments indicate that the ZCM system is both safe and has excellent tumor inhibition properties. ZCM is a novel system for CO controlled release, with significant potential to improve future cancer therapy.

## Introduction

The targeted development of cancer treatment technology—including chemotherapy, radiotherapy, and immunotherapy—has enormous potential in tumor treatment, although most such technologies are yet to be used routinely in the clinic (1–5). Among therapeutic approaches being developed, treating cancer with CO gas is both novel and practical, yet remains underexplored. High-dose CO can reduce cell protein synthesis by inhibiting cellular mitochondrial respiration and induce cancer cell apoptosis (6–8). The safety and potency of CO therapy relies on the precise release of large amounts of CO directly into the tumor (8). However, most CO-related anticancer treatments are still in their initial stages, as the gaseous nature of CO makes controlled release highly problematic.

As a CO prodrug, manganese carbonyl (MnCO) has a Fenton-like reaction with H_2_O_2_ to generate CO gas *in situ* (9). CO can then bind hemoglobin in tumor tissue, reducing its capacity for oxygen transport, causing mitochondrial damage, and thus achieving an anticancer effect without causing side effects systemically (9). Zhu and coworkers designed a novel type of CO delivery system using the immune evasion ability and tumor targeting of tumor-derived exosomes (EXO), encapsulating MnCO into exosomes by electroporation method to form the MMV system (6); this system could achieve a powerful antitumor effect in combination with low-dose radiotherapy. Both *in vivo* and *in vitro* experiments have verified the rationality of the MMV combined with RT designed by their team, and there is no inflammatory reaction and other side effects during the treatment period, which has good biological safety. Jin and his team used hollow mesoporous silica nanoparticle (hMSN) nanocarriers to effectively encapsulate MnCO and constructed a nano-drug (MnCO@hMSN) for antitumor research (10). After being internalized into the tumor tissue, the nanomedicine (MnCO@hMSN) will react with H_2_O_2_ in the tumor (a new Fenton-like reaction that releases CO gas *in situ*) to achieve antitumor effects. *In vivo* experiments have shown that within the treatment cycle. The weight of the mice did not have any abnormalities, and the tumor proliferation was significantly inhibited (10). However, in cancer cells, although intracellular H_2_O_2_ concentration can reach 50 μM, endogenous H_2_O_2_ is unable to achieve satisfactory efficiency (11–13). Insufficient H_2_O_2_ in the tumor microenvironment (TME) is a profound problem for MnCO-based cancer therapy. CPT is a natural product topoisomerase inhibitor operating *via* several mechanisms including induction of cellular DNA damage (14–16). It is also an H_2_O_2_ enhancer, inducing high levels of H_2_O_2_ in the tumor (14). This property has interested several researchers. Tang et al. overcame the lack of target-specific, high-intensity luminescence by creating a target-specific chemiluminescence strategy, where CPT was loaded into the CLDRS system, enhancing H_2_O_2_ concentration and chemiluminescence (14). However, CPT has poor delivery to tumors and low water solubility, limiting its systemic use in cancer therapy.

Use of new nanocarriers allows for the design of secure and efficient multifunctional nanoplatforms for accurate drug delivery and effective cancer treatment. As a new metal-organic framework (MOF), ZIF-8 has good drug delivery properties and biocompatibility (17–21). Compared to other metal-organic framework (MOF) materials (22–26), ZIF-8 has several unique characteristics. ZIF-8 comprises 2-methylimidazole and Zn^2+^. Zinc is the second most abundant transition metal in biology, while biogenic amino acid histidine contains an imidazole group (27). ZIF-8 has exceptional chemical and thermal stability, particularly in aqueous conditions; high specific surface area; and negligible physiological toxicity (28). Moreover, decomposition occurs easily under acidic conditions (pH 5.0–6.0), making ZIF-8 suitable for stimulus-responsive controlled drug release of payloads in the acidic tumor microenvironments (18). ZIF-8 is thus an ideal template for preparing hollow nanomaterials with adjustable sizes for small-molecule drug delivery. The ZIF-8-loaded drugs are less likely to leak during transit to their site of action, retaining pharmaceutical activity until they reach their target tumors (29, 30). Efficient delivery of CPT/MnCO using ZIF-8 nano-frameworks is expected to overcome many limitations of current CO gas therapy.

**Scheme 1:**
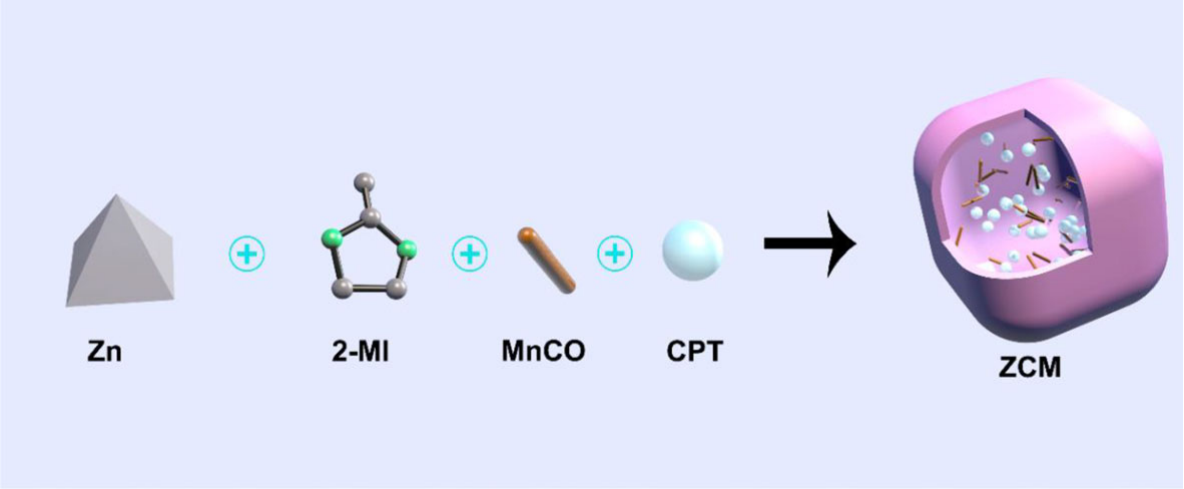
Illustration of a novel H_2_O_2_ generator for enhanced CO gas therapy.

We designed a therapeutic strategy that combines chemotherapy drug CPT and CO gas prodrug MnCO in a ZIF-8 nanocarrier with good drug delivery properties forming a composite system we named ZCM (**Scheme 1**). After intravenous injection, ZCM circulates systemically, reaching the tumor target, where it is endocytosed. Subsequently, CPT and MnCO are released in response to the acidic tumor microenvironment, where CPT also acts as a H_2_O_2_ generator increasing the concentration of H_2_O_2_ in the tumor, synergistically improving the anticancer activity of MnCO. MnCO reacts with H_2_O_2_ to generate CO *in situ*, directly damaging mitochondria. *In vitro* and *in vivo* experiments indicate that the ZCM system has a high antitumor effect and does not induce significant off-target toxicity, since ZCM does not leak MnCO. The development of ZCM highlights a powerful new approach to MOF-based nanomedicines.

## Results and Discussion

**Figure 1:**
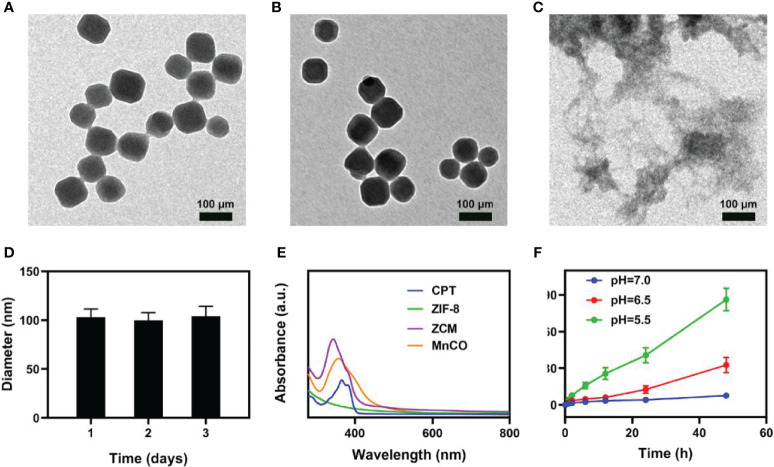
Characterization of ZCM. **(A)** TEM image of ZIF-8 and **(B)** ZCM. **(C)** TEM image of decomposed ZCM. **(D)** DLS was used to measure the hydrodynamic diameter of ZCM. **(E)** UV–vis spectra for CPT, ZIF-8, MnCO, and ZCM in PBS. **(F)** *In vitro* CPT release profile at different pH from ZCM.

ZIF-8 nanomaterials of approximately 100 nm were prepared using a simple stirring method (30). Then ZCMs were synthesized by simultaneous embedment of CPT and MnCO into ZIF-8 to form a ZCM system, as shown in **Figures 1A, B** for transmission electron microscope (TEM) images of pure ZIF-8 and ZCM. A TEM image of ZCM, as measured in an acidic environment (**Figure 1C**), suggests that ZCM can decompose in an acidic environment. The diameter of ZCM (**Figure 1D**) showed almost no changes, again indicating that ZCM was stable and did not alter due to inclusion of drugs. The absorption of CPT, ZIF-8, MnCO, and ZCM was measured using UV–vis absorption spectroscopy, with results indicating the successful preparation of ZCM (**Figure 1E**). The drug loading efficiency of CPT and MnCO in ZCM was found to be 14.6% and 17.8%. ZCM’s drug release ability was also studied (**Figure 1F**). Under neutral conditions, ZCM did not decompose and CPT was not released. At pH = 6.5, after 48 h of coculture, CPT release was approximately 30%. At pH = 5.5, CPT release was approximately 38% at 24 h; after 48 h, this had reached almost 90%, with the CPT almost completely released. As shown in **Figure S1**, the release rate of CO rapidly enhances with increasing H_2_O_2_ concentrations. This indicated that ZCM can release CO in response to the tumor microenvironment. This indicates that ZCM can help control payload release in the tumor environment, which is expected to alter the tumor microenvironment and realize CO gas therapy.

**Figure 2:**
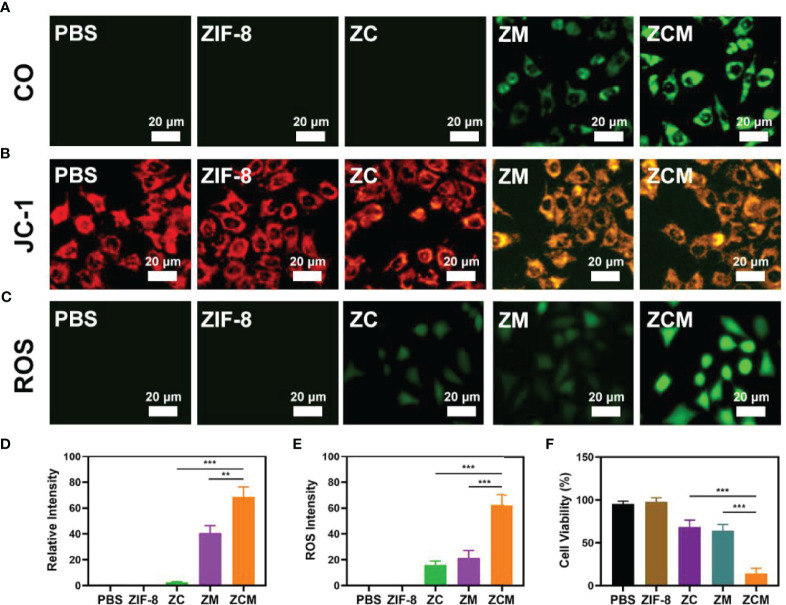
*In vitro* synergetic therapeutic effects of the ZCM. **(A–C)** CO (FL-CO-1), JC-1 (green for JC-1 monomer and red for JC-1 aggregate), and ROS (DCFH-DA) fluorescence images under different treatments. **(D)** Quantification analysis of CO **(A)** based on the relative intensity of counts of at least 100 cells per treatment group (n = 3). **(E)** Fluorescence intensity of ROS in **(C)** based on the relative intensity of counts of at least 100 cells per treatment group (n = 3). **(F)** The survival of CT26 cells with different treatments. **p < 0.01, ***p < 0.005; Student’s t-test.

Our ZCM system performs well, and we are actively conducting *in vitro* antitumor trials. Although MnCO greatly inhibits tumors, the CO yield generated by the H_2_O_2_-MnCO reaction is affected by the H_2_O_2_ concentration in the tumor: at 50–100 μM, this is higher than in ordinary cells, but is still limited. Therefore, it is necessary to increase the tumor H_2_O_2_ concentration. Our system contains the chemotherapeutic drug CPT, which produces H_2_O_2_. We verified the ability of ZCM to produce CO; see **Figures 2A, D**. The control group and single ZIF-8 or ZC showed no fluorescence, suggesting a low intracellular CO concentration. ZM containing MnCO shows weak fluorescence. The green fluorescence of the ZCM treatment group was the strongest, since CPT can generate H_2_O_2_ in TME, promoting the reaction between MnCO and H_2_O_2_. Changes in mitochondrial membrane potential (MMP) in tumor cells were monitored using the JC-1(5,5′,6,6′-tetrachloro-1,1′,3,3′-tetraethyl-imidacarbocyanine) probe method. Typically, JC-1 dye accumulates in the mitochondria, where it aggregates, producing a red fluorescence. However, in damaged mitochondria where MMP is reduced, monomeric dye is released into the cytoplasm, producing green fluorescence. **Figures 2B, E** shows the high green/red fluorescence ratio of cells treated with ZCM. This is consistent with reduced mitochondrial damage due to ZCM. Once ZCM is decomposed by the acidic environment of tumor cells, MnCO will react with H_2_O_2_ in TME to produce CO gas *in situ*. This causes serious mitochondrial damage. In addition, we measured the ROS content of different formulations: the ZCM group had high fluorescence, while that of the ZC and ZM groups was much lower (**Figure 2C**). This may be because CPT alters the tumor microenvironment and MnCO produces elevated ROS. ROS can degrade cellular protein and DNA, thus killing tumor cells (27, 31–33). An MTT assay test indicated that the cell viability of the control and ZIF-8 groups was minimally affected, while ZC alone or ZM induced moderate tumor growth inhibition (**Figure 2F**). Our ZCM system showed the greatest tumor inhibition, reaching 90%. There are significant differences when compared to other experimental groups, suggesting that ZCM-mediated increased H_2_O_2_ concentrations of TME can enhance the effect of MnCO, inhibiting tumor growth.

**Figure 3:**
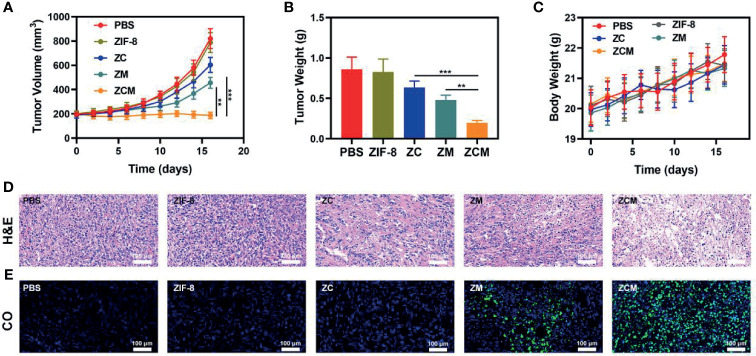
*In vivo* therapy. **(A)** Tumor volume, **(B)** weight, and **(C)** murine body weight were monitored in the five treatment groups (n = 5). **(D)** Tumor sections were stained for H&E. **(E)** Fluorescence imaging for co-localization of the tumor region to test CO production. (Blue: DAPI, green: FL-CO-1). **p < 0.01, ***p < 0.005; Student’s t-test.

**Figure 4:**
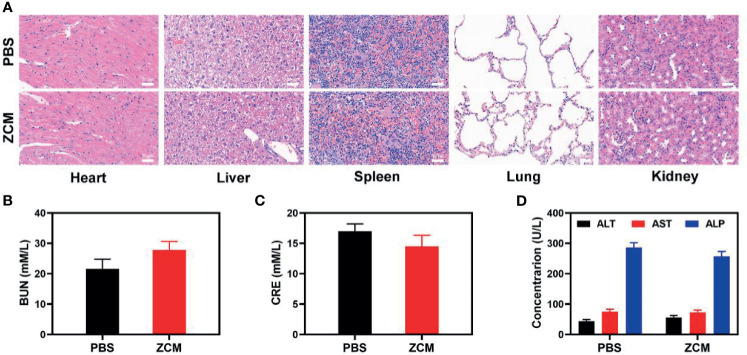
**(A)** Histopathologic examination of the tissues including the heart, liver, spleen, lung, and kidney from tumor-bearing mice after PBS or ZCM treatment. **(B)** Blood biochemistry data for kidney function marker BUN. **(C)** Blood biochemistry data including kidney function marker CRE. **(D)** Liver function markers: ALT, AST, and ALP.

We next evaluated the ZCM-mediated antitumor efficacy in mice with CT26 tumors. To investigate the principal effect of ZCM, BALB/c mice were injected subcutaneously into the right flank with 1 × 10^6^ CT26 cells. The mice were treated when the primary tumor volume reached 200 mm^3^. Tumor-bearing mice were randomly divided into five groups (five mice per group): (1) PBS; (2) ZIF-8; (3) ZC; (4) ZM; and (5) ZCM. The equivalent CPT dose was 10 mg/kg in groups 3 and 5. Treatment was conducted every 3 days for 16 days. During treatment, the tumor volumes of the control and ZIF-8 groups rose rapidly, which is shown in **Figures 3A, B**. The ZC or ZM group showed a moderate tumor suppression effect. When ZIF-8 reaches the tumor and is endocytosed, the acidic tumor microenvironment causes the ZIF-8 framework to decompose, releasing its drug payload and inducing a therapeutic effect. The ZCM system had the most marked therapeutic effect, with tumor volume growth almost entirely suppressed during treatment. During this study, no weight changes were detected in the treatment group, indicating no significant systemic toxicity (**Figure 3C**). This is crucial since many treatments have clear systemic toxicity, which significantly decreases their potential utility (34–37). We used the FL-Co-1 + PdCl_2_ fluorescence probe to detect the CO content of tumors, confirming that combining CPT and MnCO in ZCM greatly enhanced CO generation in the tumor. We took tumor tissue sections for staining. H&E, TUNEL, and Ki-67 staining (**Figures 3D, E** and **S2, S3**) confirmed there was significant cell necrosis in the ZCM group. As shown in **Figure 4**, there was likewise no inflammatory damage, and liver and kidney indexes were normal. The *in vivo* results indicate that our novel treatment achieved both good biological safety therapy and increased tumor H_2_O_2_ concentration, reinforcing the effect of ZCM with profound CO-based therapy.

## Conclusion

We designed a novel H_2_O_2_ generator ZCM to realize enhanced CO gas therapy. Encapsulated chemotherapeutic agents CPT and MnCO can increase the concentration of H_2_O_2_ in the tumor microenvironment driving CO gas therapy, with an enhanced ability to induce tumor cell apoptosis. The *in vitro* and *in vivo* results indicate our system has an excellent tumor inhibition effect. Our ZCM system exhibited no clear toxicity during treatment. As non-toxic materials have better application potential and will also reduce the pain of patients during treatment. Although many materials have good antitumor effects, their systemic toxicity significantly affects the application value. We will explore the biological application of our combination of MnCO and other novel nanotechnology vehicles, optimizing our treatment plan.

## Data Availability Statement

The raw data supporting the conclusions of this article will be made available by the authors, without undue reservation.

## Ethics Statement

The animal study was reviewed and approved by the Institutional Animal Care and Use Committee of Wuhan University.

## Author Contributions

Conceived and designed the experiments: YL, ZL, and WZ. Performed the experiments: YL, ZL, DJ, YW, KZ, and CL. Contributed reagents/materials/analysis tools: NZ, DJ, YW, KZ, and CL. Revised and polished the article: NZ, YL, ZL, DJ, YW, KZ, and CL. All authors contributed to the article and approved the submitted version.

## Funding

This work was supported by the National Natural Science Foundation of China (81901771) and Zhongnan Hospital of Wuhan University Science, Technology and Innovation Seed Fund, Project znpy2019022.

## Conflict of Interest

The authors declare that the research was conducted in the absence of any commercial or financial relationships that could be construed as a potential conflict of interest.

## Publisher’s Note

All claims expressed in this article are solely those of the authors and do not necessarily represent those of their affiliated organizations, or those of the publisher, the editors and the reviewers. Any product that may be evaluated in this article, or claim that may be made by its manufacturer, is not guaranteed or endorsed by the publisher.
