# Supplementary figures and images for: A Novel H2O2 Generator for Tumor Chemotherapy-Enhanced CO Gas Therapy
Source: Front Oncol. 2021 Sep 21;11:738567. doi: 10.3389/fonc.2021.738567 (PMC8496405; doi:10.3389/fonc.2021.738567)

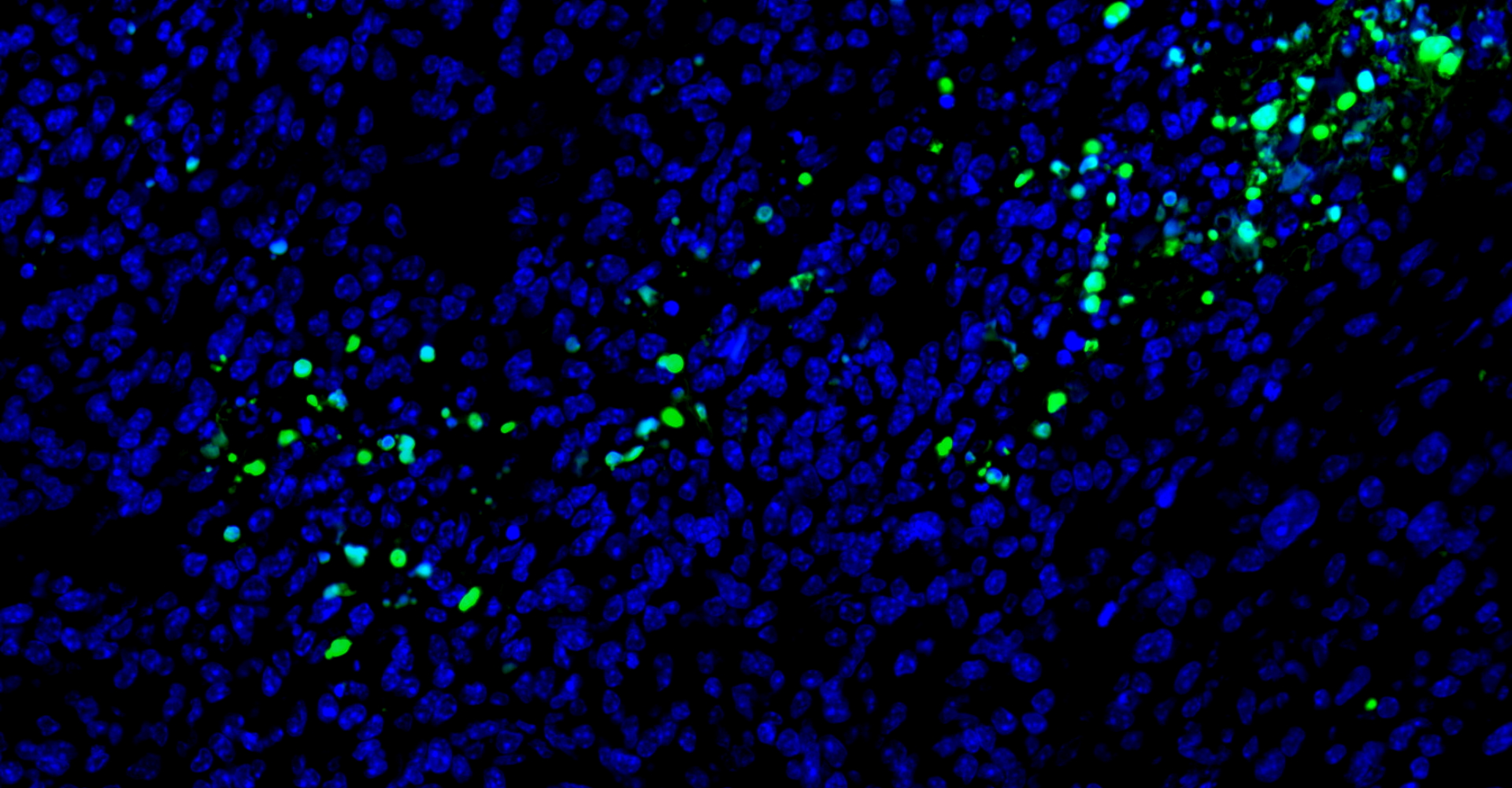

Supplement: Supplementary file 1 [file Image_1.jpeg]

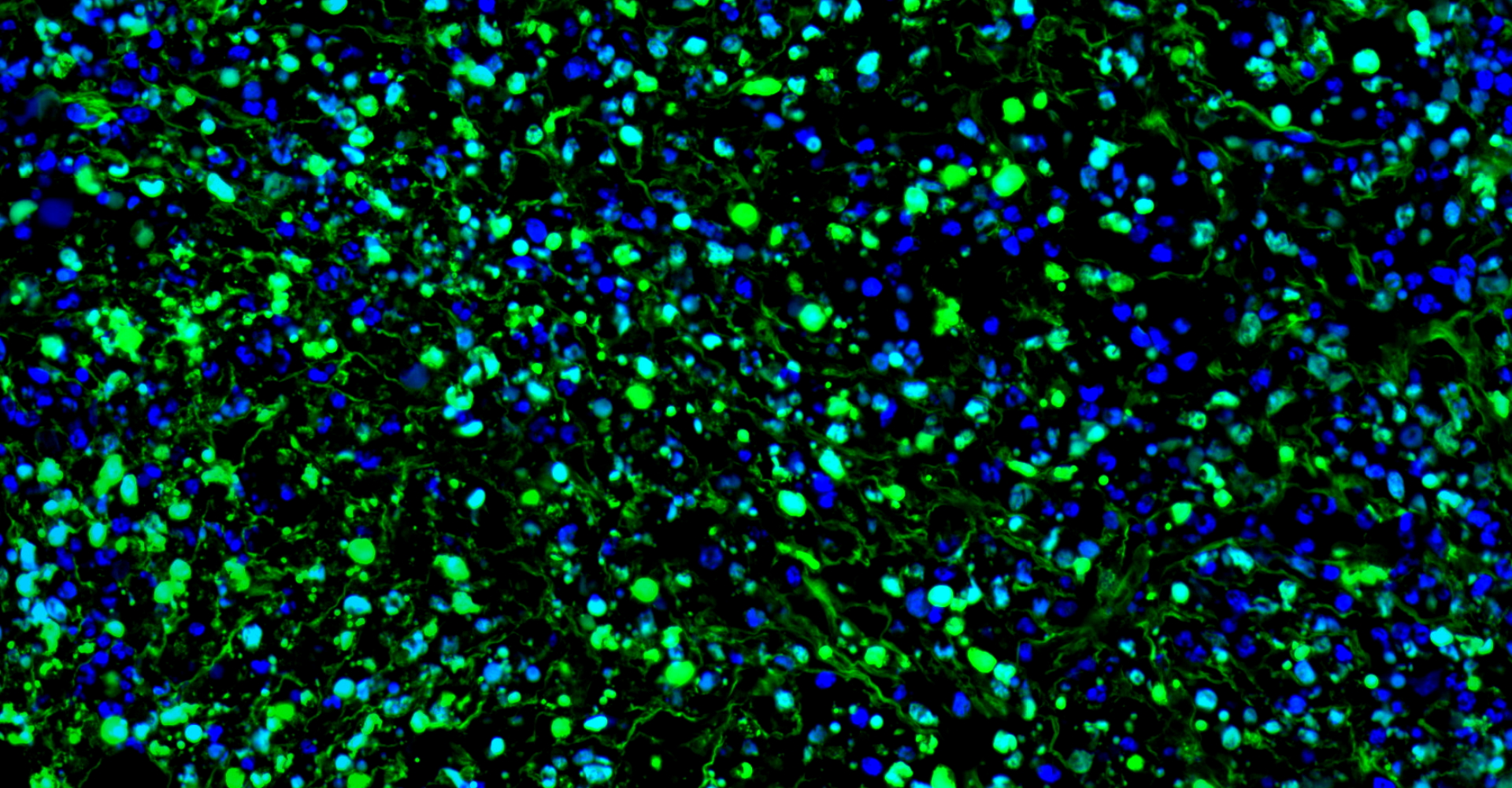

Supplement: Supplementary file 2 [file Image_2.jpeg]

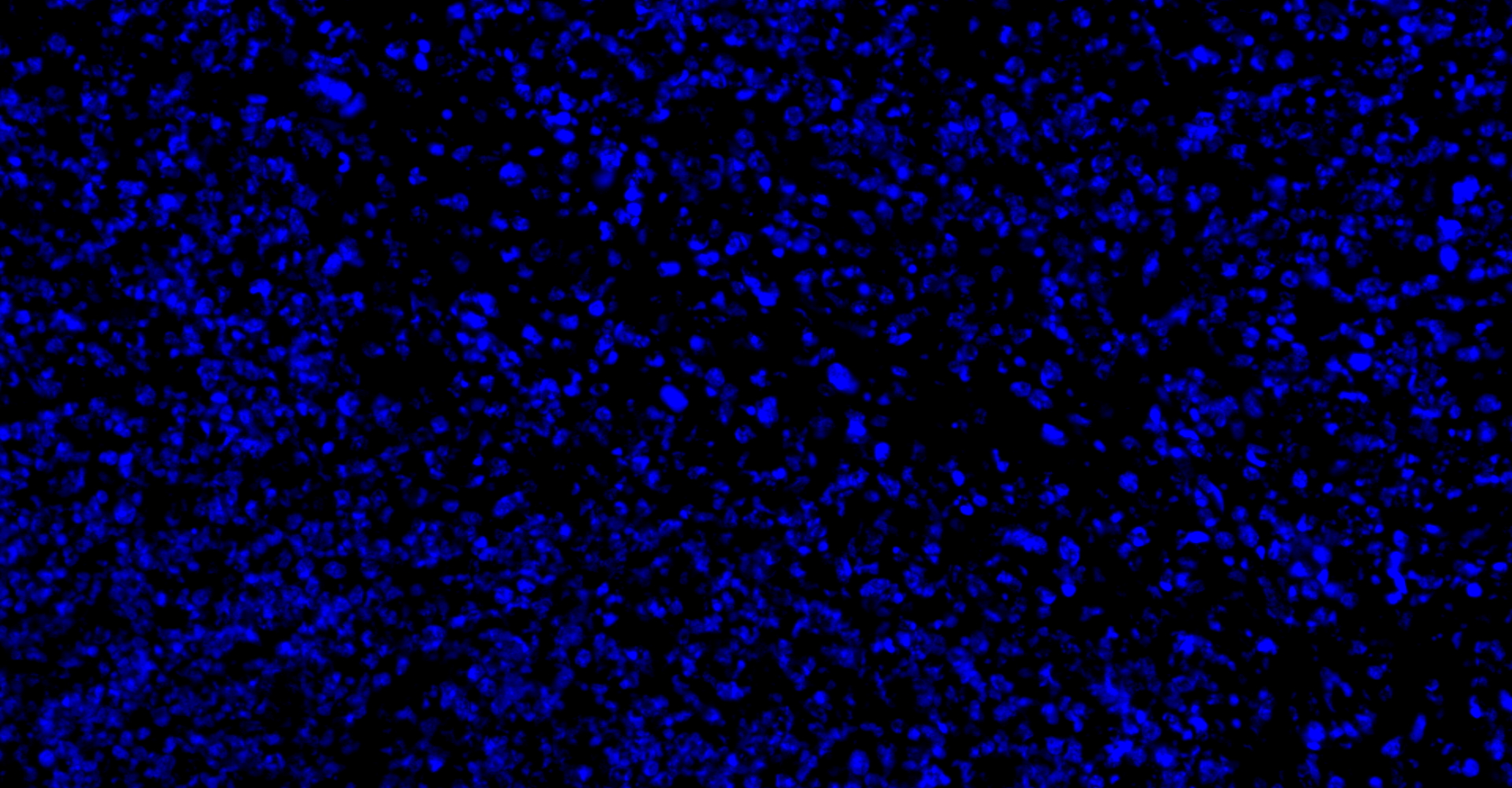

Supplement: Supplementary file 3 [file Image_3.jpeg]

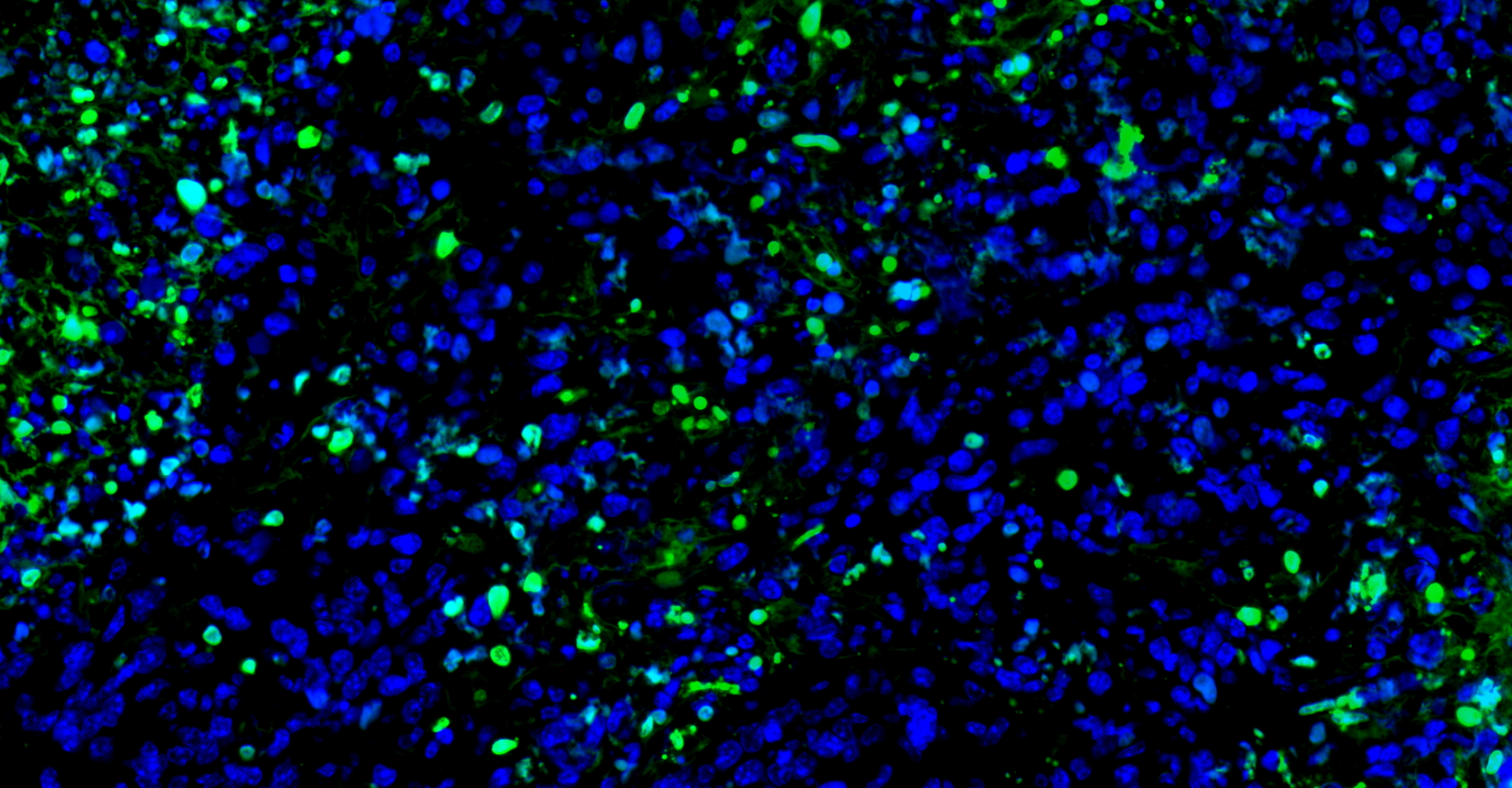

Supplement: Supplementary file 4 [file Image_4.jpeg]

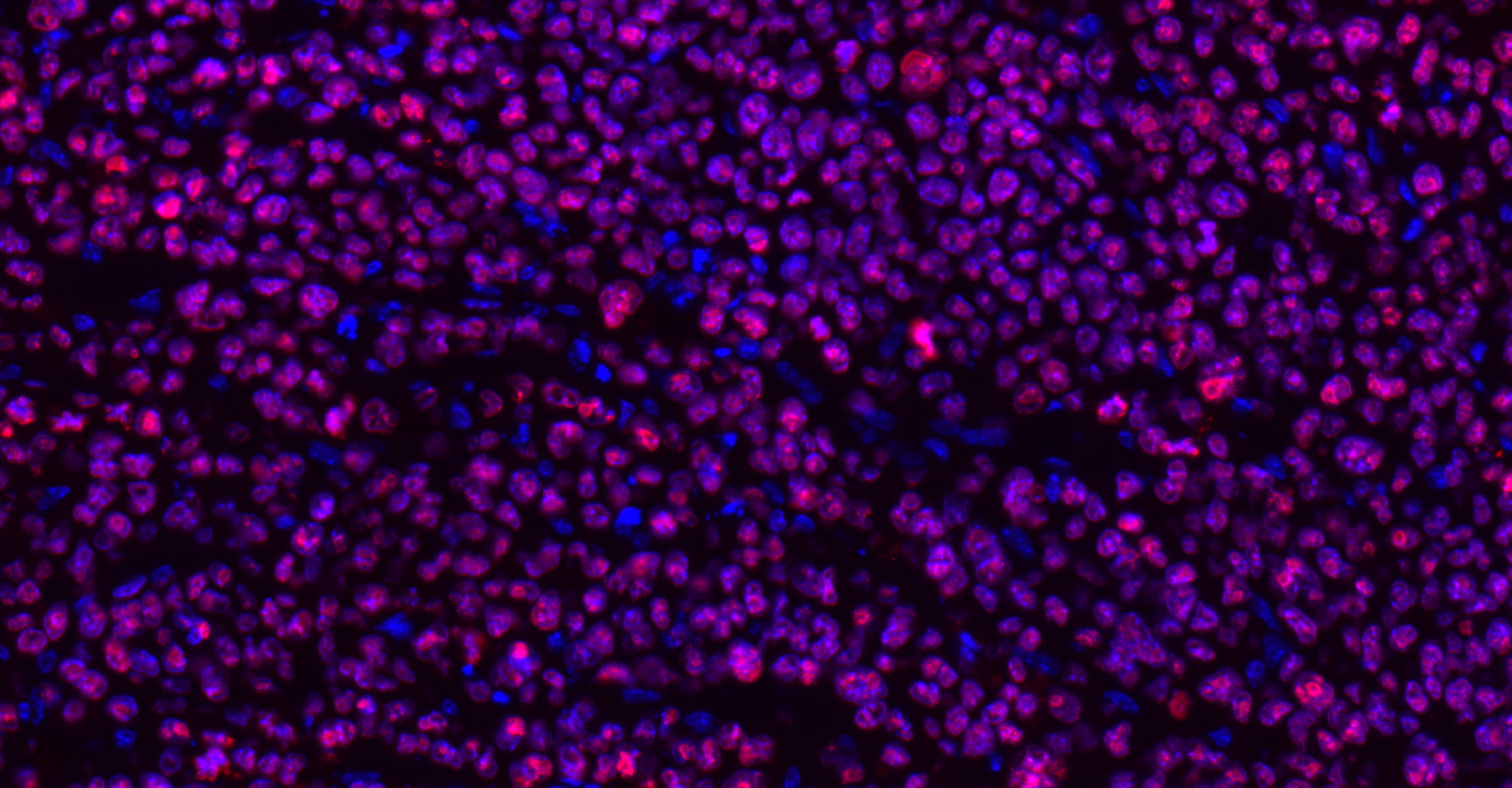

Supplement: Supplementary file 5 [file Image_5.jpeg]

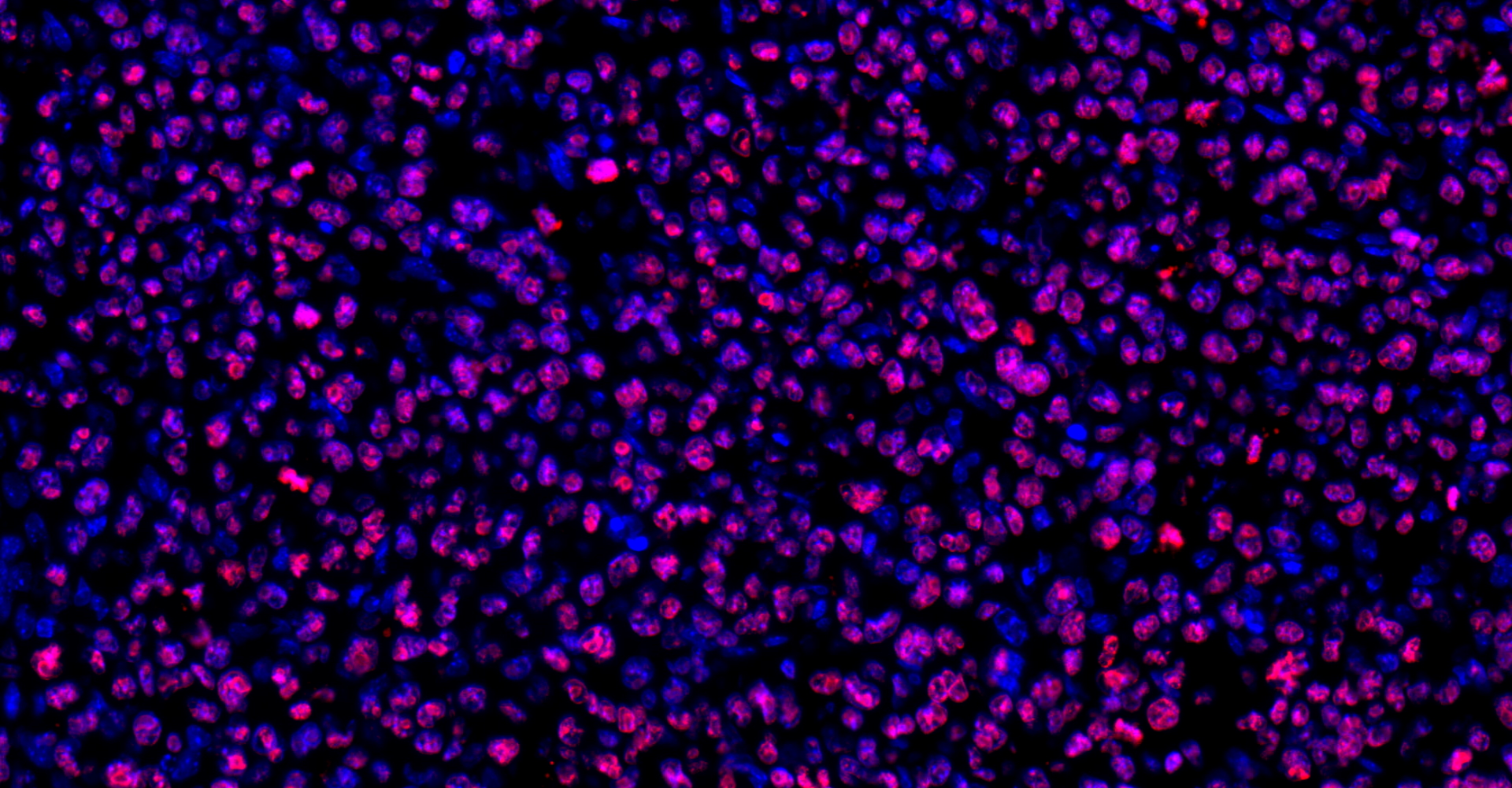

Supplement: Supplementary file 6 [file Image_6.jpeg]

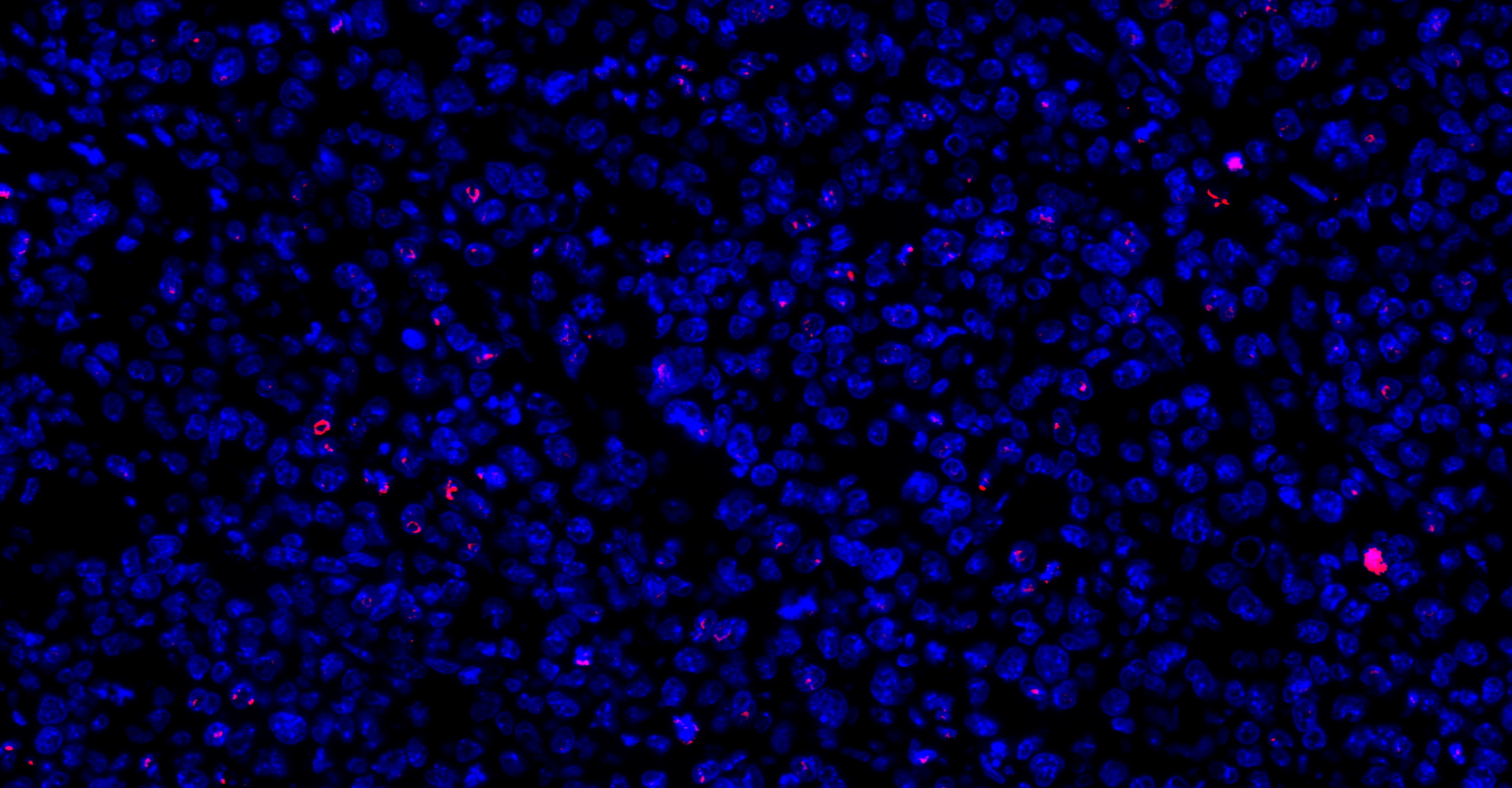

Supplement: Supplementary file 7 [file Image_7.jpeg]

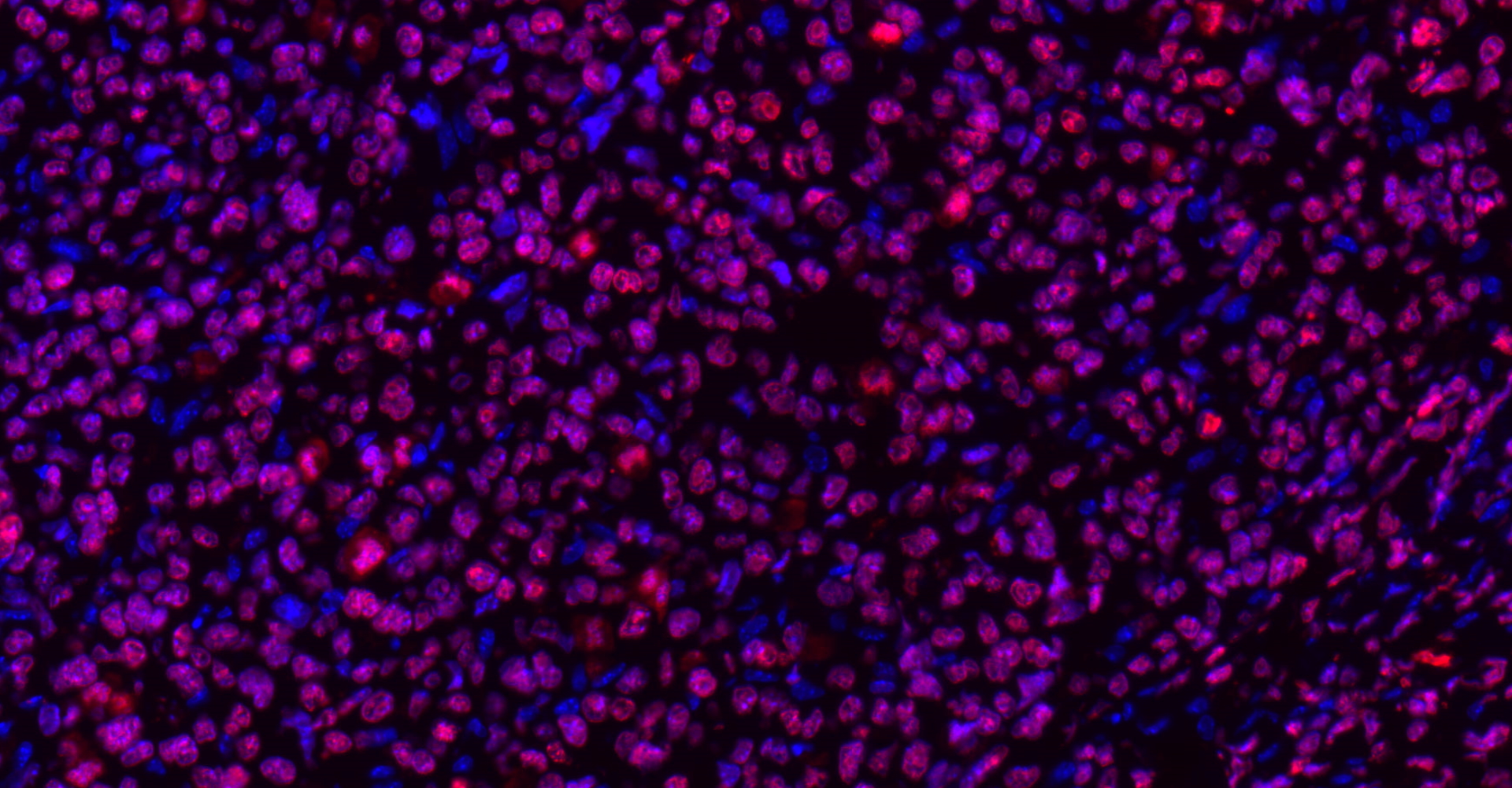

Supplement: Supplementary file 8 [file Image_8.jpeg]

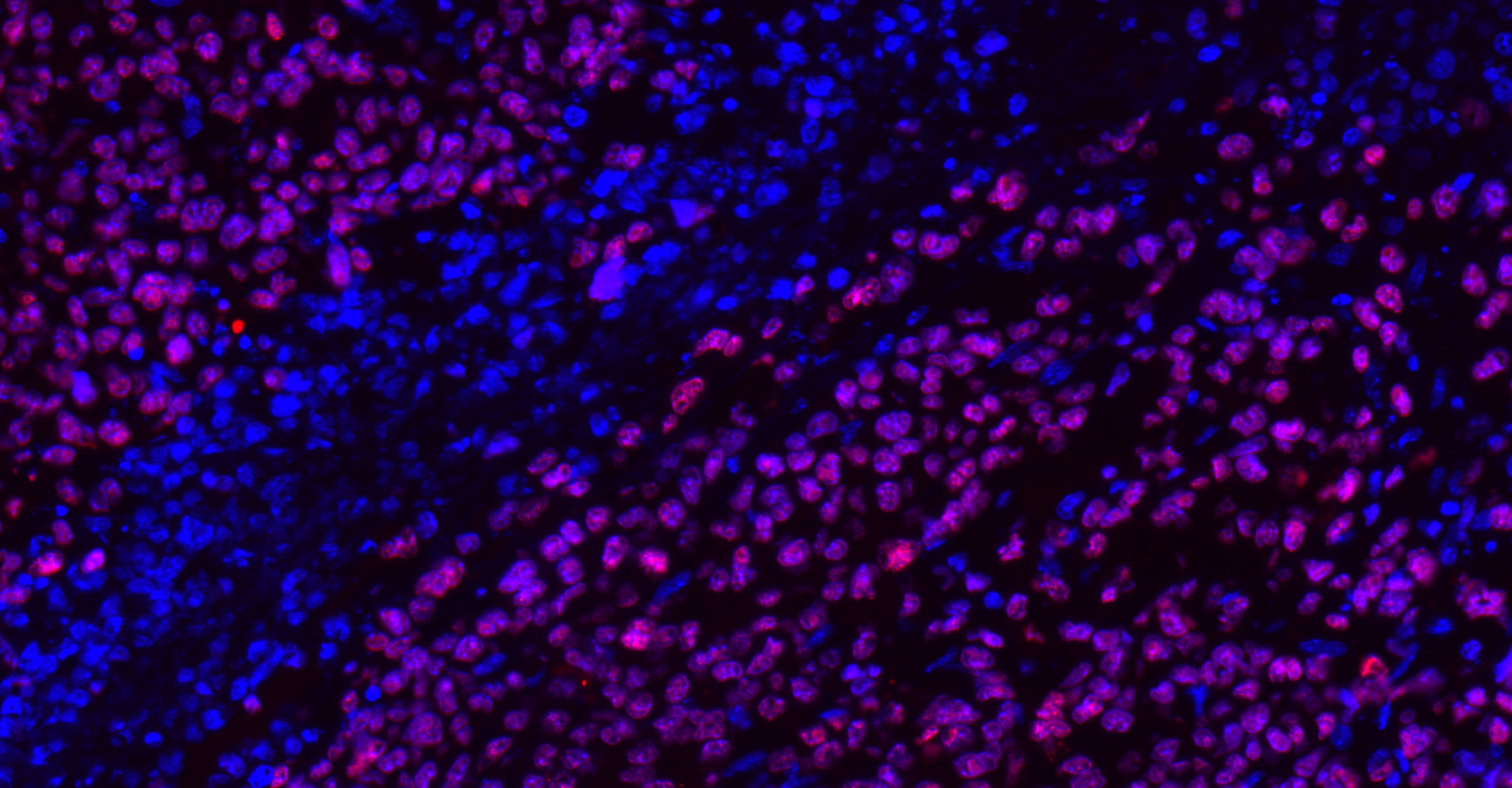

Supplement: Supplementary file 9 [file Image_9.jpeg]
