# Supplementary material for: A Novel H2O2 Generator for Tumor Chemotherapy-Enhanced CO Gas Therapy
Source: Front Oncol. 2021 Sep 21;11:738567. doi: 10.3389/fonc.2021.738567 (PMC8496405; doi:10.3389/fonc.2021.738567)
Supplement: Supplementary file 10 [file DataSheet_1.docx]

**MATERIALS AND METHODS**

**Materials**

Camptothecin (CPT) was purchased from Aladdin Reagent (Shanghai, China).  2-methylimidazole was purchased from Macklin Reagent (China). Reactive oxygen species assay kit, CCK-8 Cell Proliferation and Cytotoxicity Assay Kit, 2-(4-Amidinophenyl)-6-indolecarbamidine dihydrochloride (DAPI) were purchased from Beyotime Company (China). All of the aqueous solutions were prepared using purified deionized (DI) water purified with a purification system (Direct-Q3, Millipore, USA). The other solvents used in this work were purchased from Sinopharm Chemical Reagent (China) and Aladdin-Reagent (China).

**Cell culture**

CT26 mouse colon cancer cell line cells were obtained from the Cell Bank of the Chinese Academy of Sciences and incubated in RPMI-1640 medium supplemented with 10% FBS in a humidified atmosphere at 37℃ with 5% CO_2_.

**Animal Models**

Female BALB/c aged 4-5 week were purchased from Vital River Company (Beijing, China). 100 ul of CT26 cell suspension (5×10^6^ cells per ml) were subcutaneous injected into each mouse to establish the tumor models. All animal procedures were approved by the Institutional Animal Care and Use Committee of Wuhan University.

**Preparation and Characterization of ZIF-8 and ZCM**

Synthesis of ZIF-8 Nanoparticles: The mixture of Zn (NO3)_2_·6H_2_O (100 mg) and 2-methylimidazole (1.94 g) was dissolved in 10 mL of deionized (DI) water. The white solid precipitate was separated by centrifugation and washed with deionized water. 5 mg MnCO (dissolved in DMSO) and 5 mg CPT (dissolved in DMSO) was added to DMSO solution containing10 mg ZIF-8 in a stirring condition prepare ZM, ZC and ZCM.

**Characterization of the ZCM nanoparticles**

The morphology structures of ZIF-8 and ZCM (pure condition and acid condition) nanoparticles were observed by the TEM (JEOL-2100). Uv-vis spectra of different samples were recorded by the Uv-vis spectrophotometry Lambda 35 (Perkin-Elmer). Hydrodynamic diameter and zeta potential were detected by the dynamic light scattering (Nano-ZS ZEN3600). The amount of MnCO loaded into ZCM was calculated from Inductively coupled plasma mass spectrometry (ICP-MS). Fluorescence spectrophotometer (FLS980) was used to detected the DLE of CPT in ZCM. Drug loading efficiency (DLE) = (weight of loaded drug/weight of feeding drug) × 100%.

**Drug release studies**

ZCM (10 mg) were packaged into a dialysis bag (MWCO = 3.0 kDa), and then the processed bags immersed in beakers of 10 mL PBS with different pH value (pH = 7.4 or 6.5) at 37 ℃. Then testing solution (1 mL) was collected at a different time interval (1, 2, 6, 12, 24 and 48 h), the contents of CPT released from ZCM were determined by UV–vis spectrophotometer. At the same time, 1.0 mL fresh PBS was added to the beaker.

**In vitro CO release**

The CO release from ZCM was measured according to the previous reported. Skeletal muscle horse myoglobin was dissolved completely in phosphate-buffered saline (PBS; pH=7.4) and then degassed by adding an excess of sodium dithionite (100 mM, 100 μL) and H_2_O_2_ (0, 50 and 100 μM) under N_2_ atmosphere. 200μL of ZCM were dispersed in PBS, bubbled with N2 for 5 min, and then added to the above solution. The reaction solution was immediately transferred into a UV quartz cuvette. The UV–vis spectrum of the solution was determined. The concentration of released CO was calculated as follows: C_CO_ = C_Hb_ (528.6×I_410nm_-304×I_430nm_)/(216.5×I_410nm_+442.4×I_430nm_), where C_CO_ is the concentration of released CO, C_Hb_ is the concentration of bovine hemoglobin, and I_410 nm_ and I_430 nm_ are the absorbance of the mixed solution at the wavelength of 410 nm and 430 nm, respectively.

**Fluorescence Imaging**

For determination of ROS levels via fluorescent imaging, CT26 cells were incubated for 6 h in five different groups: (1) PBS; (2) ZIF-8 ; (3) ZC; (4) ZM; (5)ZCM. The CPT concentration was 50 μg/mL in group 4 and 5. Then, the fluorescent dye, DCFH-DA (10 μmol/L), was added and co-incubated for 20 min at 37 °C. ROS level was determined by fluorescence microscopy. For the JC-1 assay, CT26 cells were co-incubated for 6 h in five different groups: (1) PBS; (2) ZIF-8 ; (3) ZC; (4) ZM; (5)ZCM. The cells were stained with JC-1 for 20 min before washing with PBS. Then, the mitochondrial damage/disruption was detected by fluorescence microscopy. For the CO levels, the generation of CO in cancer cells was measured by a CO probe system (FL-CO-1 + PdCl_2_). CT26 cells were co-incubated with or without MMV for 6 h in five different groups: (1) PBS; (2) ZIF-8 ; (3) ZC; (4) ZM; (5)ZCM. then with FBS-free medium containing 1 × 10^−6^ M of FL-CO-1 and 1 × 10^−6^ M of PdCl_2_ for 30 min. After rinsing with PBS (pH 7.4), the cells were observed by CLSM.

***In vitro* anti-cancer effect of ZCM**

The anti-tumor effect was measured by MTT assay. CT26 cells were seeded in 96-well plates at a density of 5 × 10^3^ cells per well and incubated for 12 h under normoxia condition. Afterwards, cells were incubated for 6 different groups: 1) PBS; (2) ZIF-8; (3) ZC; (4) ZM; (5) ZCM. The CPT concentration was 50 μg/mL in group 4 and 5. At the end of the incubation, 5 mg/mL MTT PBS solution was added, and the plate was incubated for another 4 h. Finally, the absorbance values of the cells were determined by using a microplate reader (Emax Precision, USA) at 570 nm. The background absorbance of the well plate was measured and subtracted. The cytotoxicity was calculated by dividing the optical density (OD) values of treated groups (T) by the OD values of the control (C) (T/C × 100%).

**Evaluation of *in vivo* CO generation**

The mice were firstly divided randomly into 5 groups (each group included 5 mice): (1) PBS; (2) ZIF-8; (3) ZC; (4) ZM; (5) ZCM. (Equivalent CPT concentration 10 mg/kg). 1×10^6^ CT26 cells suspended in 100 μL PBS were subcutaneously injected into each mouse to establish the tumor models. 100μL of ZC, ZM and ZCM (5mg/kg MnCO and 10mg/kg CPT) solution was intravenously injected into mice. 14 days after the tumor cells were implanted (the tumor volume is about 200 mm^3^ at this point), Then the fluorescent dye, FL-CO-1 and DAPI (10 μmol/L, 50 μL) was injected intratumorally 12 h after intravenous injection in all groups. Subsequently, tumors from each group were dissected. The cryosections were observed by a confocal laser scanning microscope (CLSM; IX81, Olympus, Japan).

***In vivo* antitumor study**

When tumor size of BALB/c mice reached approximately 200 mm^3^, the mice were divided randomly into 6 groups (each group included 5 mice): (1) PBS; (2) ZIF-8; (3) ZC; (4) ZM; (5) ZCM. (Equivalent CPT concentration 10 mg/kg). The treatment was conducted every 3 days for 15 days. Mice body weight and tumor volume in all groups were monitored every 2 days. A caliper was employed to measure the tumor length and tumor width and the tumor volume was calculated according to following formula. Tumor volume = tumor length × tumor width ^2^ / 2. After 14 days treatment, all the mice were sacrificed. the blood samples from these mice (≈1 mL) were collected for blood biochemistry analysis. Five main organs (heart, liver, spleen, lung and kidney) and tumors of all mice were harvested, washed with PBS, and fixed with paraformaldehyde for histology analysis. And the tumor tissues were weighed, and fixed in 4% neutral buffered formalin, processed routinely into paraffin, and sectioned at 4 μm. Then the sections were stained with hematoxylin and eosin (H&E), TUNEL and Ki-67 and finally examined by using an optical microscope (BX51, Olympus, Japan).

**Statistical analysis**

Data analyses were conducted using the GraphPad Prism 5.0 software. Significance between every two groups was calculated by the Student’s t-test. *P < 0.01, **P < 0.005, ***P < 0.001.


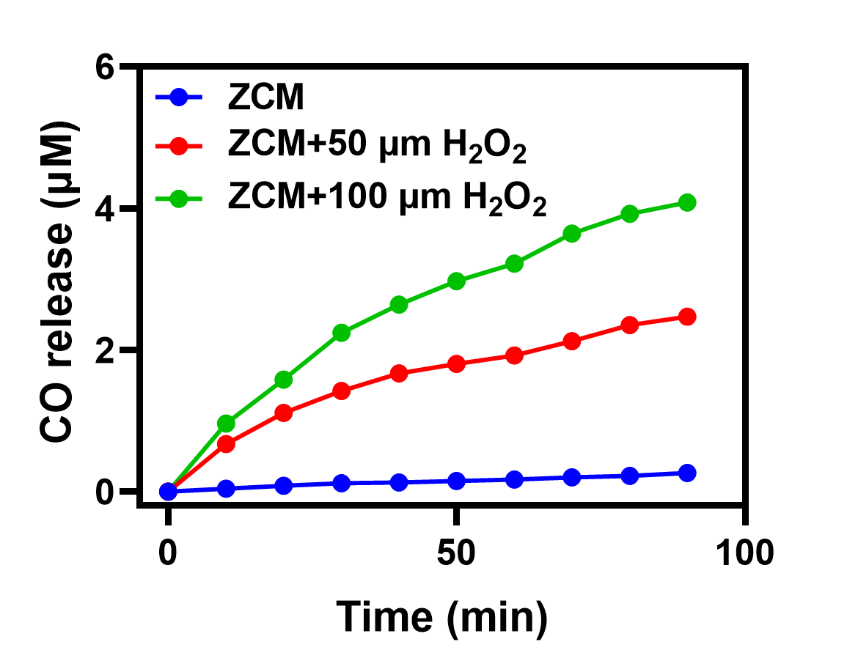


Figure S1. In vitro CO release profile at 37 ◦C from ZCM after different treatment.


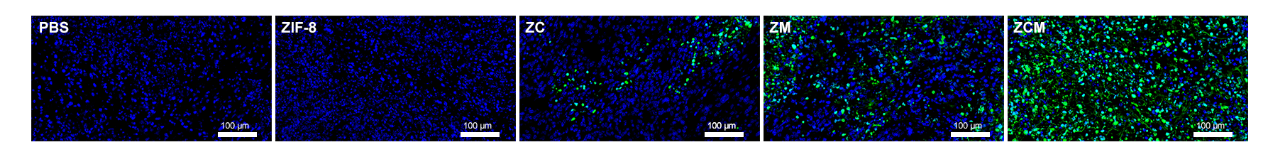


Figure S2. Tumor sections were stained for TUNEL positivity.


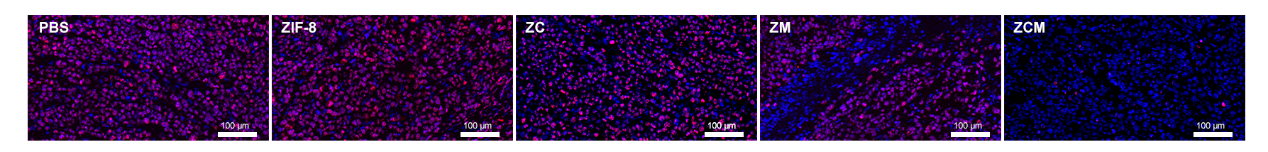


Figure S3. Tumor sections were stained for Ki-67 positivity.
